# Parvalbumin and autism: different causes, same effect?

**DOI:** 10.18632/oncotarget.14238

**Published:** 2016-12-27

**Authors:** Federica Filice, Beat Schwaller

**Affiliations:** Anatomy, Department of Medicine, University of Fribourg, Switzerland

**Keywords:** parvalbumin, ASD, Shank, VPA, perineuronal net, Neuroscience

**Figure 1:**
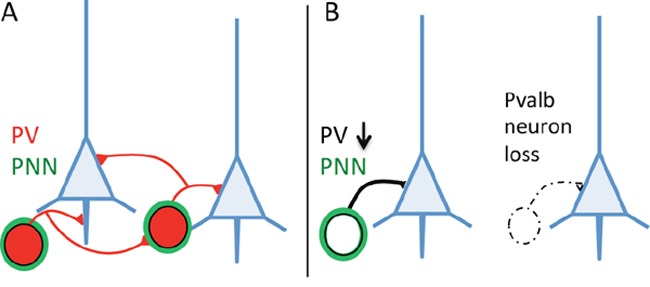
**A**. Pvalb neurons targeting pyramidal cells (blue) and other Pvalb neurons generally identified by the presence of PV (red) are surrounded by perineuronal nets (PNN; green). **B**. PV down-regulation or loss/absence of Pvalb interneurons is indistinguishable on brain sections immunostained with a parvalbumin antibody only.

Autism spectrum disorders (ASD) are multifactorial conditions, with obvious genetic, epigenetic and/or environmental risk factors implicated in the disease etiology. Identification of several gene mutations in human ASD and rodent models carrying mutations in the same candidate genes recapitulating the behavioral core symptoms of ASD -namely lack of social interaction, impaired communication and repetitive, stereotyped behaviors -helped to test hypotheses on the etiology of ASD. Discomfortingly, single gene defects account only for 1% of ASD cases [1]; thus the search of convergent pathways aimed to “reduce ASD complexity” might help to develop therapies to treat ASD. In recent years, a subpopulation of inhibitory (GABAergic) interneurons (hereafter termed Pvalb neurons) expressing the calcium-binding protein parvalbumin (PV) has gained increased attention with respect to ASD. Pvalb neurons play a key role in the coordination of neuronal networks and associated oscillations. Studies in ASD animal models [2] revealed the number of PV-immunoreactive (PV^+^) cells to be decreased, and such a decrease was also observed in post mortem brains of ASD patients [3]. The decrease of PV^+^ neurons was (implicitly or explicitly) assumed to be the result of partial loss of the Pvalb neurons (Figure 1). Since also mice with reduced (PV+/−) or absent (PV−/−) PV expression levels show behavioral deficits relevant to human ASD core symptoms [4], the question arose, whether the reduction in the number of PV^+^ neurons was the result of Pvalb neuron loss and/ or a decrease in PV expression. From a viewpoint of PV immunohistochemistry, the two possibilities are indistinguishable, requiring a second marker for the identification of Pvalb neurons: e.g. *Vicia Villosa Agglutinin* (VVA), a lectin binding to specific extracellular matrix components forming a net-like structure around Pvalb neurons (Figure 1). The number of PV^+^ or VVA-positive (VVA^+^) neurons was counted in three ASD-associated brain regions: medial prefrontal cortex, somatosensory cortex and striatum. Results in all three regions were identical: the unchanged number of VVA^+^ cells in PV+/− and PV−/− mice demonstrated that Pvalb neurons are still present and that the lower number of PV^+^ neurons (in PV+/− mice) or the absence of PV^+^ cells (in PV−/− mice) is the result of diminished/absent expression of PV in these interneurons [5]. Since the *PVALB* gene is not considered as a *bona fide* ASD risk gene (as it has never been identified in genetic screens), we analyzed knockout mouse models of two well-accepted ASD risk genes: *Shank1* and *Shank3*. Mutations in *SHANK* genes are considered as highly relevant factors for ASD. In both, Shank1−/− and Shank3B−/− mice, we observed a decrease in PV^+^ neurons in the SSC and striatum, respectively, resulting from decreased *Pvalb* mRNA and PV protein, yet without any significant changes in the number of Pvalb neurons. Most recently, Pvalb neurons were investigated in a widely recognized environmental ASD mouse model [2], i.e. in mice exposed *in utero* to valproic acid (VPA). Again the number of Pvalb neurons was unaltered, while the observed lower number of PV^+^ neurons in the striatum (-15%) was due to a decrease in *Pvalb* mRNA (-50%) and PV protein (-30%) [6]. Taken together, the down-regulation of PV in genetic (Shank1−/−, Shank3B−/−, PV+/−) and environmental (VPA) mouse ASD models, and moreover in specific brain regions provides a common link between seemingly unrelated defects causing ASD. The origin of the reported decrease in the number of PV^+^ neurons in human ASD brains [3], as well as in other mouse ASD models reported before [4] remains an open, however highly pertinent question. Although it is unquestionable that alterations of the Pvalb-neuron circuitry profoundly affect neuronal networks, ultimately resulting in/ contributing to the so-called excitation/inhibition imbalance in ASD, we consider it an absolute necessity to unequivocally distinguish between Pvalb neuron loss or reduction of PV protein levels. In the first place, this likely leads to a more substantiated interpretation of the previously reported physiological changes in mouse ASD models with lower PV^+^ neuron numbers. Evidently, loss of Pvalb neurons leads to diminished inhibition, whereas PV down-regulation increases short-term facilitation and thus “enhances” inhibition [for review see 7]. Second, if PV down-regulation, not Pvalb neuron loss, would be confirmed in other ASD models with reported lower numbers of PV^+^ neurons, then a diminution in PV and the physiological changes associated with it might represent a common convergent pathway, possibly resulting in the core ASD phenotype despite different initial causes, e.g. as a downstream consequence of mutations in ASD risk genes. This in turn might reveal PV as an emerging new target, in particular when proven that PV upregulation might rescue, or at least diminish, the ASD behavioral phenotype.
